# Trends in Manufacturer-Reported Nicotine Yields in Cigarettes Sold in the United States, 2013–2016

**DOI:** 10.5888/pcd17.200205

**Published:** 2020-11-25

**Authors:** Nicole Kuiper, Ellen M. Coats, Tamara N Crawford, Doris G. Gammon, Brett Loomis, Clifford H. Watson, Paul C. Melstrom, Rene Lavinghouze, Todd Rogers, Brian A. King

**Affiliations:** 1 Office on Smoking and Health, Centers for Disease Control and Prevention, Atlanta, Georgia; 2 Center for Health Analytics, Media, and Policy, RTI International, Research Triangle Park, North Carolina; 3 Division of Laboratory Sciences, National Center for Environmental Health, Centers for Disease Control and Prevention, Atlanta, Georgia

## Abstract

**Introduction:**

A gradual reduction of cigarette nicotine content to nonaddictive levels has been proposed as an endgame strategy to accelerate declines in combustible tobacco smoking. We assessed manufacturer-reported nicotine yield in cigarettes sold in the United States from 2013 to 2016.

**Methods:**

We merged machine-measured nicotine yield in cigarette smoke and pack characteristics obtained from reports filed by tobacco manufacturers with the Federal Trade Commission for 2013–2016 with monthly Nielsen data on US cigarette sales. Manufacturer-reported, sales-weighted, average annual nicotine yield was assessed, as were nicotine yield sales trends by quartile: markedly low (0.10–0.60 mg/stick), low (0.61–0.80 mg/stick), moderate (0.81–0.90 mg/stick), and high (0.91–3.00 mg/stick). Trends in overall, menthol, and nonmenthol pack sales, by nicotine yield quartiles over the study period and by year, were determined by using Joinpoint regression.

**Results:**

During 2013–2016, average annual sales-weighted nicotine yield for all cigarettes increased from 0.903 mg/stick (95% CI, 0.882–0.925) in 2013 to 0.938 mg/stick (95% CI, 0.915–0.962) in 2016 (*P* < .05). For menthol cigarettes, yield increased from 0.943 mg/stick in 2013 (95% CI, 0.909–0.977) to 1.037 mg/stick in 2016 (95% CI, 0.993–1.081), increasing 0.2% each month (*P* < .05). Most pack sales occurred among high (41.5%) and low (30.7%) nicotine yield quartiles. Cigarette sales for the markedly low quartile decreased by an average of 0.4% each month during 2013–2016 (*P* < .05).

**Conclusion:**

During 2013–2016, manufacturer-reported, sales-weighted nicotine yield in cigarettes increased, most notably for menthol cigarettes. Continued monitoring of nicotine yield and content in cigarettes can inform tobacco control strategies.

**Summary**

**What is already known on this topic?**

Previous research shows that cigarette nicotine yields in the United States increased from 1997 to 2005 because of cigarette design modifications.

**What is added by this report?**

Our study found that the manufacturer-reported average annual nicotine yield of menthol cigarettes increased from 2013 to 2016 in the United States, and sales for all cigarettes in the lowest nicotine yield quartile declined. Nicotine yields of some top-selling brands fluctuated during this period, and nearly 20% of products sold lacked reported nicotine yields.

**What are the implications for public health practice?**

Monitoring tobacco sales for product availability and consumer preference is important to reduce smoking-related disease and death in the United States.

## Introduction

Nicotine is the ingredient in cigarettes that causes addiction (1). Reducing the nicotine content of cigarettes was first proposed in 1994 as a strategy to reduce the risk of addiction from cigarettes (2). Research has subsequently shown that considerable reductions in nicotine content in cigarettes can result in decreased toxicant exposure and reduced smoking behavior and dependence (3–6). In 2014, the US Surgeon General proposed the gradual reduction of cigarette nicotine content as a potential endgame strategy to accelerate declines in combustible tobacco smoking (7).

The 2009 Family Smoking Prevention and Tobacco Control Act gave the US Food and Drug Administration (FDA) authority to regulate cigarettes (8). This includes the ability to reduce nicotine content in cigarettes but prohibits FDA from completely removing nicotine from cigarettes (8). In 2018, FDA requested data to inform a potential tobacco product standard to lower nicotine content in cigarettes to minimally addictive or nonaddictive levels (9,10). Exact thresholds for minimal and no addictiveness are uncertain; however, the average cigarette contains approximately 10 mg of nicotine, and research suggests nicotine content would have to be very low (eg, 0.05 mg) to avoid compensatory behaviors that happen at higher levels (eg, 0.3 mg) and to lead to substantial cessation and reduced toxicant exposure (2,6).

The relationship between nicotine content in a cigarette stick and actual yield to the user is complex because of the potential for compensating behaviors by users to regulate their nicotine intake. Smokers modulate puffing and inhalation in response to variations in yield (11). Nonetheless, a 2018 simulation model suggested that lowering nicotine content of cigarettes to minimally addictive levels in the United States would reduce smoking prevalence to 1.4%, prevent 16 million people from initiating smoking, and avoid 2.8 million tobacco-related deaths by 2060 (12).

Studies have found an association between cigarette nicotine yield and product design characteristics (13) and that cigarette nicotine yields increased from 1994 to 2004 (13,14). Machine‐generated measures of nicotine yield remain the most widely available method for assessing and comparing nicotine yields across cigarette brands. However, no study has assessed recent trends in cigarette sales by nicotine yield. Therefore, we assessed trends in manufacturer-reported cigarette sales by nicotine yield from 2013 to 2016.

## Methods

### Manufacturer-reported cigarette nicotine yield data

Data on manufacturer-reported nicotine yield (mg/stick) in cigarettes manufactured and sold annually in the United States during calendar years 2013 through 2016 were obtained from the US Federal Trade Commission (FTC) (15). All nicotine yield data are manufacturer-reported and not independently measured to confirm the average nicotine intake when a person smokes.

Information in the FTC data set is provided at the Universal Product Code (UPC) (ie, barcode) level for each cigarette variety. In addition to nicotine yield, the data set also provides information on the cigarette brand (eg, Marlboro, Camel), subbrand (eg, Marlboro Southern Blend, Camel Crush), package color (eg, white), length (eg, king, long), filter status (eg, filtered), menthol status (menthol or nonmenthol), and pack type (soft pack or hard pack).

### Retail sales data

We obtained UPC-level retail sales data for cigarettes from The Nielsen Company (Nielsen). These data included sales that occurred from January 13, 2013, through January 7, 2017, in convenience stores, mass merchandisers, supermarkets, drug stores, dollar stores, club stores, and military commissaries in the contiguous continental United States; Nielsen sales data were not available for Hawaii and Alaska. Sales were reported in approximately monthly (4-week) aggregates from January 13, 2013, through January 7, 2017. Because more than half of the days in the final 4-week period occurred in calendar year 2016, this period was considered part of 2016. Therefore, the study period is referred to as 2013–2016 hereinafter. The data set also included UPC-level descriptive variables similar to those provided in the FTC data (eg, brand, subbrand, filter status), and information about package size (eg, number of sticks per pack, number of packs per UPC).

### Combining measures of manufacturer-reported nicotine yield with cigarette sales

Although UPC codes were provided in both the FTC and Nielsen data, these codes are not in consistent formats; thus, matching items in the 2 data sets required that the data be grouped and matched by characteristics to define a unique product, including brand, subbrand, package color, length, filter status, menthol status, and pack type. Before matching, each data set was cleaned to ensure that characteristics were formatted consistently across data sets.

In some cases, multiple items in the FTC data had identical product characteristics but varying nicotine yield values. To determine a single nicotine yield value for each product in a given year, mean nicotine yield was calculated. In cases where the nicotine yield for a product was missing in 1 or more years, but present in other years of data, mean nicotine yield for that product across years without missing data was imputed for years with missing nicotine yield. Following this imputation, 57 of the 742 unique products (7.7%) present in the FTC data were missing nicotine yield information in all years.

In the Nielsen data, package color information was missing or contained outdated cigarette pack descriptions for some items. Since 2009, as part of the Family Smoking Prevention and Control Act, tobacco companies are prohibited from marketing products with terms that suggest reduced harm, such as “light” and “mild.” However, cigarette product characteristics for 43% of the items in the Nielsen UPC data have not been updated to reflect this change (eg, product description still is listed as light). Therefore, when possible, 2 independent analysts conducted internet searches to determine package color by using available product descriptions, because package color often is used to communicate product characteristics that are no longer allowed (16). The analysts’ code was compared and reconciled, resulting in a final package color determination for 309 unique products in the Nielsen data. For example, the package color “gold” (previously called “light” until 2009 when such descriptors were deemed deceptive and federal law prohibited their use) was assigned to nonmenthol Marlboro cigarette products with no distinguishing subbrand when the listed product strength description was light. Finally, 1 product with a nicotine yield of 6.6 mg/stick, more than double the next-highest nicotine yield value, was determined to be an outlier and excluded. The resulting analytic data set included nicotine yield values for 325 unique cigarette products, reflecting 80.2% of cigarette pack sales present in the Nielsen data.

### Analysis

Cigarette unit sales were standardized by using package size information to represent the equivalent units of a single pack of 20 cigarettes (hereinafter, “pack sales”). Pack sales within each cigarette product were aggregated to create monthly product-level pack sales. Annual measures of nicotine yield then were matched to each monthly sales observation by product. We excluded products in the Nielsen data for which there was no matching nicotine yield information in the FTC data (19.8% of pack sales).

Quartiles of manufacturer-reported nicotine yield were calculated by year for products in the FTC data with reported nicotine yield. Interquartile ranges for 2013–2014 and 2016 were as follows: 0.10–0.60 mg/stick, markedly low; 0.61–0.80 mg/stick, low; 0.81–0.90 mg/stick, moderate; and 0.91–3.00 mg/stick, high. In 2015, however, ranges for moderate and high categories were defined slightly differently (0.81–0.94 mg/stick for moderate and 0.94–3.00 mg/stick for high), because of changes in reported nicotine yields among top-selling brands. By year, products were classified into the aforementioned quartiles based on the manufacturer-reported nicotine yield. We use the category titles of markedly low, low, moderate, and high to describe the quartiles and not as classification recommendations for levels of nicotine or to minimize the addictiveness of cigarettes. Quartiles were used to better understand the range of products offered on the market each year by manufacturers instead of nicotine levels in cigarette sticks, which were not available for most products.

To assess manufacturer-reported cigarette nicotine yield during 2013–2016, we calculated monthly and annual average nicotine yield for cigarettes sold for all products and by flavor (menthol vs nonmenthol), weighting by pack sales. The weighting was conducted such that nicotine yield for products with larger sales volume more heavily influenced the average. We then used *t* tests to identify differences in sales-weighted average annual nicotine yield and trends in average monthly nicotine yield for all products, and by flavor, from 2013 through 2016. Sales data do not have consumer information, so it is not possible to assess individual consumer behavior; however, the advantage of a sales-weighted average is that it allows comparisons by year and between product types, taking into account the products that consumers are actually purchasing. The percentage of pack sales by nicotine quartile (eg, pack sales for high-yield cigarettes as a proportion of overall pack sales) was calculated overall and for menthol and nonmenthol cigarettes. Finally, Joinpoint software, version 4.5.0.1 (National Cancer Institute) was used to test for trends in overall, menthol, and nonmenthol pack sales by nicotine yield classification (markedly low, low, moderate, and high) over the full study period and by year (17). Joinpoint analyses controlled for autocorrelation and used a log-linear functional form to measure the average monthly percentage change in sales (18). All tests were significant at *P* < .05.

## Results

### Manufacturer-reported nicotine yields: sales-weighted averages

Manufacturer-reported nicotine yields in the analytic data set ranged from 0.1 to 3.0 mg/stick. Although we saw no consistent increase in monthly sales-weighted average nicotine yield among all cigarettes during 2013–2016, average annual sales-weighted nicotine yield was significantly higher in 2016 (0.938 mg/stick; 95% CI, 0.915–0.962) compared with 2013 (0.903 mg/stick; 95% CI, 0.882–0.925) (*P* < .05) (Table 1). For menthol cigarettes, sales-weighted average nicotine yield increased by 0.2% each month (*P* < .05) during 2013–2106, and average annual sales-weighted nicotine yield increased from 0.943 mg/stick in 2013 (95% CI, 0.909–0.977) to 1.037 mg/stick in 2016 (95% CI, 0.993–1.081). We found no monthly or annual changes in sales-weighted manufacturer-reported nicotine yield among nonmenthol cigarettes.

**Table 1:** Sales-Weighted Average of Annual Changes in Manufacturer-Reported Nicotine-Yield Levels and Average Monthly Percentage Change (AMPC), by Flavor Category, United States, 2013–2016

Flavor Category	Sales-Weighted Average Annual Nicotine Yield (mg/stick) (95% CI)				Difference in Means: 2013 vs 2016 (95% CI)	2013–2016 AMPC
	2013	2014	2015	2016		
Overall	0.903 (0.882 to 0.925)	0.915 (0.893 to 0.936)	0.839 (0.820 to 0.859)	0.938 (0.915 to 0.962)	0.035^a^ (0.003 to 0.067)	0.1 (0.0 to 0.2)
Menthol	0.943 (0.909 to 0.977)	0.974 (0.939 to 1.009)	0.885 (0.858 to 0.911)	1.037 (0.993 to 1.081)	0.094^a^ (0.039 to 0.150)	0.2^a^ (0.0 to 0.4)
Nonmenthol	0.889 (0.861 to 0.917)	0.892 (0.865 to 0.919)	0.822 (0.795 to 0.848)	0.899 (0.872 to 0.925)	0.010 (-0.028 to 0.048)	0.0 (−0.1 to 0.1)

a Significant difference (*P* < .05) in means between 2013 and 2016 using a *t* test.

## Pack sales by manufacturer-reported nicotine yield and flavor

In all years except 2015, most pack sales overall occurred among high nicotine yield cigarettes, ranging from 39.3% in 2013 to 54.2% in 2016, followed by low, moderate, and markedly low nicotine yield cigarettes (Figure). In 2015, cigarettes with a moderate nicotine yield comprised the largest annual market share (36.5%), followed by cigarettes with low (33.6%), high (21.3%), and markedly low (8.6%) nicotine yields. The change in 2015 was due to lower reported nicotine yield levels among the top-selling Marlboro and Newport products, which resulted in reclassification of these products from the high to the moderate nicotine yield category, as compared with 2014 and 2016. This reclassification in 2015 affected both menthol and nonmenthol products (Table 1). Similarly, menthol cigarette sales were more concentrated among high-yield cigarettes in each year except 2015. In 2016, 75% of menthol cigarette sales occurred among high-yield cigarettes (Figure), which was the highest proportion of any nicotine quartile in any year.

**Figure:**
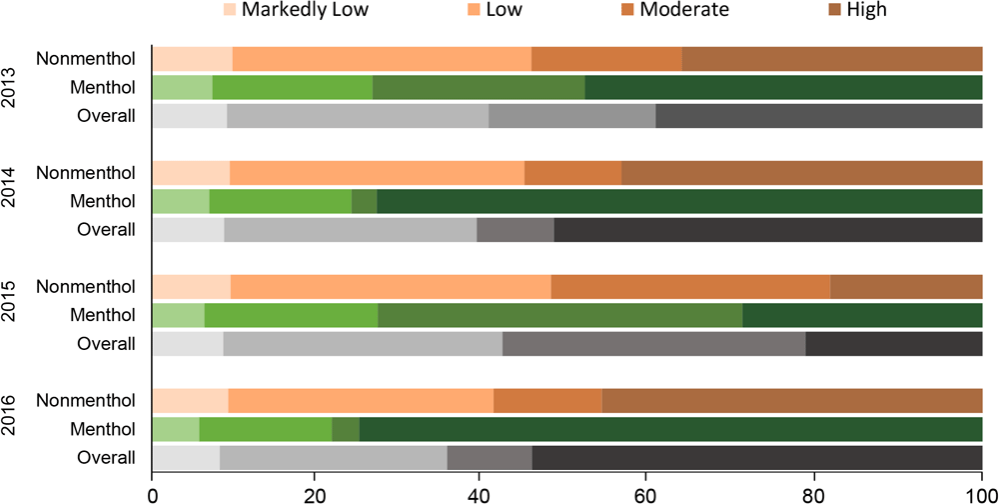
Annual market share of cigarette pack sales by quartiles of manufacturer-reported nicotine yield and flavor, United States, 2013–2016. The distribution of cigarette sales in each period by nicotine quartile indicates that cigarette sales were generally concentrated among moderate-nicotine and high-nicotine products. Application of sales weights to the calculation of average nicotine yield accounts for this skewness by allowing the averages to more closely reflect more commonly sold products. Graded coloration reflects reported nicotine content for nonmenthol, menthol, and overall pack sales by quarter, with darker coloration reflecting greater nicotine content.

**Table:** 

Year	Type	Markedly Low, %	Low, %	Moderate, %	High, %
2016	Overall	8.2	27.4	10.2	54.2
	Menthol	5.7	16.0	3.3	75.0
	Nonmenthol	9.2	32.0	13.0	45.8
2015	Overall	8.6	33.6	36.5	21.3
	Menthol	6.4	20.8	43.9	28.9
	Nonmenthol	9.5	38.6	33.6	18.3
2014	Overall	8.7	30.4	9.3	51.6
	Menthol	6.9	17.1	3	72.9
	Nonmenthol	9.4	35.5	11.7	43.4
2013	Overall	9.1	31.5	20.1	39.3
	Menthol	7.3	19.3	25.6	47.9
	Nonmenthol	9.7	36.0	18.1	36.2

### Average monthly cigarette pack sale trends

Among cigarette products with markedly low nicotine yield, pack sales decreased by an average of 0.4% each month during 2013–2016 (*P* < .05), with menthol and nonmenthol sales decreasing significantly each month (by 0.4% for menthol and 0.3% for nonmenthol) (Table 2). Pack sales among all other nicotine yield quartiles did not significantly change when the full study period was considered. However, menthol cigarette sales with moderate nicotine yield decreased by an average of 6.3% each month during 2013–2016 (*P* < .05). Within-year analysis of sales by nicotine yield showed stable trends among menthol cigarettes with markedly low and low yield during 2013, 2014, and 2015 (Table 2). However, sales in 2016 among cigarettes with both markedly low and low nicotine yield decreased by 0.5% per month on average (*P* < .05). Menthol sales remained stable and nonmenthol sales decreased by an average of 0.5% each month for markedly low yield cigarettes and 0.6% each month for low yield cigarettes. Sales of cigarettes with moderate nicotine yield increased significantly by 0.3% per month in 2015 and decreased significantly by 0.7% per month in 2016, with moderate yield menthol sales significantly decreasing by 4.9% each month on average during 2016. Although there were no significant trends in pack sales of cigarettes with high nicotine yield during 2013–2016, significant positive average monthly percentage change were observed during 2014 (0.4%) and 2015 (0.6%). Pack sales of menthol cigarettes with high nicotine yield significantly increased by an average of 1.2% each month during 2013, 0.7% each month during 2014, and 1.0% each month during 2015. Pack sales of nonmenthol cigarettes with high nicotine yield significantly increased by 0.4% each month on average during 2015–2016.

**Table 2:** Annual Trends in Average Monthly Cigarette Pack Sales by Manufacturer-Reported Nicotine Yield Quartiles and Percentage Menthol and Nonmenthol Cigarettes, United States, 2013–2016

Nicotine Yield	2013		2014		2015		2016		2013–2016
	AMPC (95% CI)	Average Monthly Pack Sales (SE)^a^	AMPC (95% CI)	Average Monthly Pack Sales (SE)^a^	AMPC (95% CI)	Average Monthly Pack Sales (SE)^a^	AMPC (95% CI)	Average Monthly Pack Sales (SE)^ a^	AMPC (95% CI)
**Quartile 1 – Markedly low^b^**									
Overall	−0.2 (−1.1 to 0.7)	63,579,140 (678,159)	−0.2 (−0.6 to 0.2)	59,588,311 (558,431)	0.2 (−0.3 to 0.6)	58,409,755 (487,946)	−0.5^c^ (−0.9 to −0.1)	54,181,080 (463,419)	−0.4^c^ (−0.6 to −0.1)
Menthol, %	0.0 (−1.0 to 1.1)	21.7	−0.2 (−0.4 to 0.0)	21.9	0.0 (−0.5 to 0.5)	20.6	−0.4 (−1.8 to 1.0)	20.0	−0.4^c^ (−0.6 to −0.2)
Nonmenthol, %	−0.3 (−1.2 to 0.6)	78.3	−0.2 (−0.6 to 0.3)	78.1	0.2 (−0.2 to 0.7)	79.4	−0.5^b^ (−0.8 to −0.3)	80.0	−0.3^c^ (−0.6 to −0.1)
**Quartile 2 – Low^b^**									
Overall	−0.2 (−0.7to 0.2)	221,105,737 (2,155,057)	0.2 (−0.1 to 0.5)	207,929,199 (2,032,613)	0.2 (−0.3 to 0.8)	227,700,089 (1,852,371)	−0.5^c^ (−0.8 to −0.3)	180,817,939 (1,602,682)	−0.4 (−0.8 to 0)
Menthol, %	−0.1 (−0.3 to 0.2)	16.5	0.6 (−0.2 to 1.3)	15.5	0.3 (−0.1 to 0.7)	17.3	−0.5 (−1.2 to 0.3)	16.7	−0.4 (−1.0 to 0.3)
Nonmenthol, %	−0.3 (−0.9 to 0.3)	83.5	0.1 (−0.3 to 0.5)	84.5	0.2 (−0.3 to 0.6)	82.7	−0.6^c ^(−0.8 to −0.4)	83.3	−0.4 (−0.8 to 0)
**Quartile 3 – Moderate^b^**									
Overall	−0.1 (−0.5 to 0.3)	141,341,463 (1,863,590)	0.3 (−0.4 to 1.0)	63,637,596 (670,662)	0.3^c^ (0.1 to 0.5)	247,161,803 (2,288,170)	−0.7^c^ (−1.4 to 0)	67,503,509 (763,155)	−1.9 (−5.0 to 1.4)
Menthol, %	0.4 (–0.8 to 1.6)	34.3	−0.1 (–0.5 to 0.2)	9.0	0.5 (−0.1 to 1.0)	33.6	−4.9^c^ (−5.2 to −4.6)	9.2	−6.3^c^ (−11.2 to −1.1)
Nonmenthol, %	−0.3 (−0.9 to 0.3)	65.7	0.3 (−0.4 to 1.1)	91.0	0.3 (–0.1 to 0.7)	66.4	−0.3 (–1.0 to 0.5)	90.8	−1.0 (–3.2 to 1.2)
**Quartile 4 – High^b^**									
Overall	0.6 (−0.1 to 1.3)	276,229,914 (3,228,275)	0.4^c^ (0.2 to 0.6)	352,640,588 (3,289,022)	0.6^c^ (0.2 to 1.1)	144,300,780 (1,496,944)	0.3 (−0.2 to 0.7)	358,165,417 (3,939,838)	0.7 (−0.8 to 2.2)
Menthol, %	1.2^c^ (0.8 to 1.7)	32.8	0.7^c^ (0.3 to 1.1)	39.0	1.0^c^ (0.7 to 1.4)	37.9	0.0 (−0.5 to 0.6)	39.7	1.1 (−0.8 to 2.9)
Nonmenthol, %	0.3 (−0.2 to 0.8)	67.2	0.2 (−0.3 to 0.7)	61.0	0.4^c^ (0 to 0.8)	62.1	0.4^c^ (0.3 to 0.6)	60.3	0.4 (−0.9 to 1.7)

Abbreviation: AMPC, average monthly percentage change; SE, standard error.

a Average monthly pack sales and SEs are shown for the overall sales for each nicotine quartile. The menthol and nonmenthol figures are percentages of the overall sales in each category, and thus no SE is provided.

b Nicotine yield classifications correspond to the calculated quartiles. Quartile ranges for 2013–2014 and 2016 were as follows: 0.10–0.60 mg/stick, markedly low; 0.61–0.80 mg/stick, low; 0.81–0.90 mg/stick, moderate; and 0.91–3.00 mg/stick, high. In 2015, however, ranges for moderate and high categories were defined slightly differently as 0.81–0.94 mg/stick, moderate, and 0.94–3.00 mg/stick, high, because of changes in reported nicotine yields among top-selling brands.

c *P* < .05.

## Discussion

Average annual sales-weighted cigarette nicotine yield increased about 4% during 2013–2016, and the average annual nicotine yield increased among menthol cigarettes about 10% during the same period (from 0.943 mg/stick to 1.037 mg/stick). These findings are consistent with previous research documenting an 8.5% increase in overall manufacturer-reported cigarette nicotine yield during 1997–2008, which was approximately double the length of time of our study (13). Furthermore, during 1994–2004, the sales-weighted average nicotine yield increased 4.4% for menthol cigarettes (from 0.90 mg/stick to 0.94 mg/stick) (14). That study also found that price and average nicotine yield per cigarette were highly correlated for menthol but not nonmenthol cigarettes. This finding suggests that as prices increased and cigarette sales decreased, smokers of menthol cigarettes compensated more aggressively than nonmenthol smokers to make up for the decreased number of cigarettes consumed by purchasing fuller flavor (ie, higher nicotine yield) products as opposed to lower nicotine yield products (14). Any compensation technique could lead to sustained nicotine levels, and could lead to higher exposure to tar and nicotine for those switching to a higher nicotine brand (14).

Our findings also show that during 2013–2016, sales declined for cigarettes in the lowest nicotine yield quartile. Meanwhile, except for the fluctuation in the nicotine yield classification of top-selling products in 2015, sales of high yield nicotine cigarettes increased. This is consistent with previous research showing that cigarette nicotine yields in the United States increased during 1994–2004 (13,14). This previous increase was attributable to tobacco manufacturers increasing nicotine in the tobacco stick and to other design modifications (13). Design features previously shown to explain nicotine yield in smoke are concentration of nicotine in the tobacco stick, filter ventilation, and the number of puffs per cigarette (13). This previously increasing trend in cigarette nicotine yield underscores the importance of continued monitoring of cigarette sales and topographical studies on the extent to which modifications in design and yield may relate to population exposure and use (13).

Similar to previously observed trends toward increasing nicotine yield, our study’s findings show that in all years except 2015, from 39% to 54% of pack sales were high nicotine yield cigarettes, followed by low, moderate, and markedly low yield. In 2015, moderate nicotine yield cigarettes had the largest market share because of a change in manufacturer-reported nicotine yield among top-selling brands (ie, manufacturers reported high yields in 2014 and 2016 and moderate yields in 2015 for the same top-selling products). It is unknown whether these represent actual changes in nicotine yields for these brands for just 1 year or whether reporting errors in 1 or more years could be a factor. Additionally, although not as substantial as the average monthly percentage changes in high yield menthol cigarettes during 2013–2015, increases in average monthly sales of nonmenthol cigarettes with high nicotine yields in 2015 and 2016 also suggest that users may have responded to design changes in nicotine yields. Machine‐generated measures remain the most widely available method for assessing and comparing nicotine yields across cigarette brands (13). However, manufacturer reporting of nicotine yields is generally considered unreliable, particularly given that the FTC method was not designed to, nor can it, predict users’ varying levels of exposure to nicotine (19). Manufacturers have also attempted to circumvent the accuracy of the method (20). For example, tobacco manufacturers introduced filters with small holes located in front of where the cigarette attached to the measuring machine, which resulted in reduced nicotine yield measurements (20).

Any potential regulation of cigarettes would likely focus on nicotine levels in the cigarette itself, and not specifically machine-reported yield. However, strategies to address nicotine content, such as lowering levels in cigarettes, could also influence yield. Lowering nicotine content levels in the cigarette stick has the potential to benefit public health, as long as users do not compensate with changes in tobacco use behavior that can increase exposure to harmful constituents to maintain nicotine level. For example, in a clinical study, users of cigarettes with high nicotine content who switched to cigarettes with low (0.3 mg) nicotine content exhibited compensating behavior (eg, smoking more) and increased exposure to toxicants; however, when they switched to very low nicotine content cigarettes (0.05 mg), researchers observed decreases in cigarette intake, exhaled carbon monoxide, and exposure biomarker levels (eg, tobacco-specific nitrosamines such as NNAL [4-(methylnitrosamino)-1-(3-pyridyl)-1-butanol], NNN (N-Nitrosonornicotine) (6). Moreover, switching to cigarettes with 0.05 mg nicotine content provided better relief of withdrawal symptoms for people trying to quit than did using nicotine lozenges, suggesting that addressing non-nicotine aspects of addiction can contribute to successful cessation efforts (6). These and other clinical studies (20–22) suggest that nicotine content would have to be very low (eg, 0.05 mg) to avoid compensatory behaviors that happen at higher levels (eg, 0.3 mg) (6), and the degree of compensatory behaviors is likely dependent on level of addiction (23). Although results of the current study suggest that a large proportion of cigarettes with relatively low nicotine yield were purchased during 2013–2016, the lowest reported nicotine yield in our study was 0.10 mg, which is well above actual nicotine content levels that clinical studies indicate could lead to substantial cessation and reduced exposure to toxicants (2,6,12).

Our study has limitations. First, although FTC data included nicotine yield information for most cigarette products with high sales volume, they did not include information for all products. Overall, 19.8% of pack sales in the Nielsen database were excluded; some products (1.6% of pack sales) were listed in the FTC data, but no nicotine yield value was provided, and other products (18.1% of pack sales) were entirely missing. It is unknown whether these products with missing nicotine yield could be higher nicotine cigarettes because nicotine levels were relatively low compared with what has been studied in the literature (2). Previous research matching machine-reported nicotine yield data to scanner sales data had similar rates of missing nicotine yields, reportedly because Nielsen tracks more cigarette products than the FTC (14). Second, Nielsen’s projection methods are proprietary and do not include online or tobacco specialty stores; nonetheless, these data have been used extensively for tobacco industry monitoring, and cigarette sales online and in tobacco specialty stores are minimal in the United States (24). Third, manufacturer-reported nicotine yields do not take into account smokers’ compensating behaviors, such as puffing more deeply or covering ventilation holes (19,23,25), or other product design characteristics accounting for the variability in levels of tar, nicotine, and carbon monoxide (26). Recent changes in manufacturer-reported nicotine yields during 2013–2016 in the most popular brands, which resulted in nicotine yield quartiles changing in 2015, suggest that other design changes may have occurred. Fourth, we used national-level data in our analysis and thus were unable to assess regional differences in trends. Finally, it was not possible to examine design characteristics other than nicotine yield from machine-measured smoke, including stick nicotine content and concentration, filter ventilation, and non-nicotine components that influence addiction. This is important given that measurements of tar and nicotine yields using the FTC method do not offer smokers meaningful information on the amount of tar and nicotine they will receive from a cigarette (26). Of note, nicotine stick values were unavailable for this study, and any potential regulatory standard would likely focus on nicotine levels in the tobacco stick, and not specifically machine-reported yield (9). Thus, continued monitoring of the full scope of cigarette design characteristics is warranted, especially with regard to characteristics that could affect user exposure but are not adequately reflected through the analysis of nicotine yield.

In conclusion, average sales-weighted, manufacturer-reported nicotine yields in menthol cigarettes have continued to rise. Though many consumers are opting for cigarettes with lower nicotine yields, the lowest reported nicotine yield cigarette was 0.10 mg; though difficult to directly compare with nicotine stick content, this level is likely to be substantially higher than the threshold of 0.05 mg of nicotine content that research suggests could lead to substantial cessation and reduced exposure to toxicants (2,6,12). Thus, regulatory efforts to reduce nicotine to nonaddictive levels could be effective, though evidence suggests that other successful cessation strategies would continue to be important for those using very low nicotine cigarettes (27,28). This aligns with the 2020 US Surgeon General’s Report, which concluded that evidence is suggestive but not sufficient to infer that cigarettes with very low nicotine content can reduce smoking and nicotine dependence and increase smoking cessation when full-nicotine cigarettes are readily available. Moreover, the effects of low-nicotine cigarettes on cessation may be further strengthened in an environment in which conventional cigarettes and other combustible tobacco products are not readily available (29). Given variations in tobacco product use and exposure to tobacco marketing, including for menthol cigarettes, tobacco product standards may also have the potential to reduce tobacco-related health disparities based on age, sex, race/ethnicity, socioeconomic status, and sexual orientation (30). Continued monitoring of tobacco sales for product availability, by nicotine yield, nicotine content, and consumer preference, can inform evidence-based tobacco control strategies and regulatory efforts to diminish the addictiveness of cigarettes and reduce smoking-related disease and death.
